# Canalicular laceration (cheese wiring) with a silicone tube after endoscopic dacryocystorhinostomy: when to remove the tube?

**DOI:** 10.3205/oc000124

**Published:** 2019-10-30

**Authors:** Umut Karaca, Hakan Genc, Gulsah Usta

**Affiliations:** 1 Isparta Suleyman Demirel University, Faculty of Medicine, Department of Ophthalmology, Isparta, Turkey; 2 Gulhane Military Medical Academy, Department of Ophthalmology, Ankara, Turkey; 3 Gulhane Military Medical Academy, Department of ENT, Ankara, Turkey; 4 Kırıkkale University, Department of Ophthalmology, Kırıkkale, Turkey

**Keywords:** dacryocystorhinostomy, silicone tube, canalicular laceration, removal time

## Abstract

**Objective:** To discuss the removal time of a nasolacrimal silicone tube stent by reporting three cases with canalicular laceration due to prolonged indwelling of the stent.

**Methods:** This study involved three cases of nasolacrimal duct obstruction treated by endoscopic dacryocystorhinostomy with silicone tube insertion.

**Results:** The mean indwelling time of the silicone tube was 9.3 months and all of the patients had lower canalicular laceration near the common canaliculus. One patient presented with a complaint of canthal pain, blurred vision, and epiphora while the other two patients reported no complaint. The nasal endoscopic examination revealed a narrow fibrotic ostium below the medial concha. The silicone tubes were removed.

**Conclusions:** To increase the success rate of the operation, the removal time for nasolacrimal silicone tubes after dacryocystorhinostomy is also important. Our findings indicate that physicians should be aware of the potential problems related to prolonged intubation.

## Introduction

Symptomatic acquired nasolacrimal duct obstruction is the main etiological factor of epiphora in elderly adults. External dacryocystorhinostomy (Ex-DCR) is the gold standard treatment method for distal nasolacrimal system obstructions and endonasal dacryocystorhinostomy is also commonly performed [1], [2], [3]. After the introduction of endoscopic dacryocystorhinostomy (En-DCR), the procedure has become increasingly popular among ophthalmologists to treat nasolacrimal duct obstruction [4].

En-DCR has many benefits, such as shorter operation and hospitalization time and lack of facial scars. However, it has the foremost disadvantage of the lower success rate compared to Ex-DCR. Surgeons have developed different techniques and materials to enhance the success rate of En-DCR [5], [6].

Despite controversies in relation to stenting after operation, silicone stents are widely applied to prevent re-obstruction of the reformed rhinostomy site and have a success rate of 75–91% [7], [8], [9]. Although there are many studies indicating the removal of stents from one week up to six months, the appropriate removal time period has not yet been standardized [10], [11].

Here, we report three cases of nasolacrimal duct obstruction that have been treated by endoscopic dacryocystorhinostomy with silicone tube insertion. The mean indwelling time of the silicone tube was 9.3 months, and all the patients presented with upper and lower canalicular laceration near the common canaliculus. To the best of our knowledge, this type of complication in silicone tubes has not been reported in the literature yet.

## Methods

This is an observational study of three cases with nasolacrimal duct obstruction that have been treated by endoscopic dacryocystorhinostomy with silicone tube insertion. 

### Case 1 

**Figure 1:**
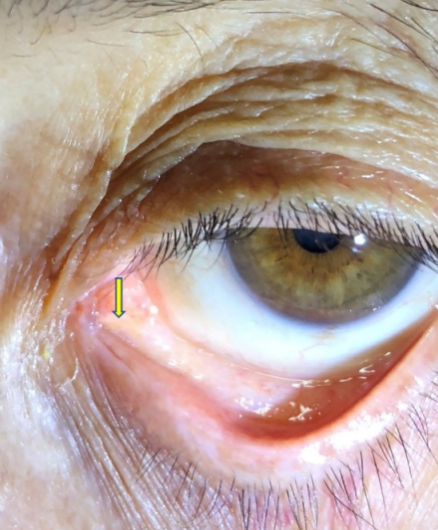
Canalicular laceration (arrow) after silicone tube extraction

A 69-year-old female presented with epiphora in her left eye that had started two years before. She had been treated with En-DCR and silicone tube intubation eight months before. Upon initial visit to our clinic, detailed ophthalmic examination and nasal endoscopy revealed an open lacrimal passage with a narrow fibrotic nasolacrimal window and proper placement of the nasolacrimal tube. A lower canalicular laceration of approximately 0.5–0.6 cm had occurred due to the silicone tube (Figure 1 (Fig. 1)). The tube was removed by nasal endoscopy and identified as a standard-type nasolacrimal intubation tube with a 0.8 mm (20 G) diameter. On the follow-up examination one week after tube removal, the patient reported no new symptoms. 

### Case 2

A 45-year-old woman was treated with En-DCR and silicone tube intubation due to her complaint of epiphora and blurred vision. The silicone tube used for the operation was a 0.90 mm x 4.5 cm (20 G diameter) angled DCR set (M04.4000, Meran Medikal, Istanbul, Turkey). She did not attend the follow-up session at the third postoperative month. At the following examination at the ninth month, a lower canalicular laceration was detected near the common canaliculus. Nasal endoscopy revealed fibrotic nasolacrimal window and shift of the silicone tube to the posterior nasal cavity. The silicone tube was removed during the endoscopic examination. 

### Case 3

**Figure 2:**
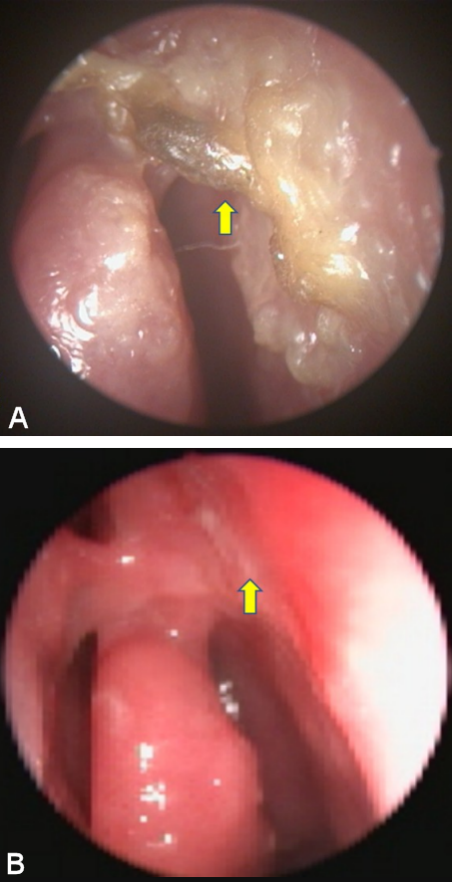
A) Intranasal silicone tube and fibrotic tissue (arrow). B) Intranasal synechia and narrow ostium (arrow).

A 56-year-old female presented with medial canthal pain, epiphora, and blurred vision 11 months after En-DCR with silicone tube intubation. A lower canalicular laceration was detected due to the silicone tube (20 G) and there were intranasal fibrotic mucosal synechiae and only a small ostium in the medial conchae region on nasal endoscopic examination (Figure 2 (Fig. 2)). The silicone tube was removed and the pain regressed during the follow-up, but epiphora persisted. Nasolacrimal lavage from the upper punctum revealed insufficient drainage during dacryocystorhinostomy.

All three cases had undergone dacryocystorhinostomy combined with bicanalicular intubation to improve the postoperative success rate. The location of the stent was appropriate and the stent was well tolerated by the patients without any allergic complication. Although the nasal ostium was open during the early postoperative period, osteal stricture was seen after seconder fibrotic healing of nasal mucosa (Figure 2B (Fig. 2)). Displacement of the tube narrowing the ostium and prolonged intubation may have been the cause of secondary stent tension and cheese wiring in all three cases.

## Discussion

In recent years, En-DCR has become the most preferred method for the treatment of nasolacrimal duct obstruction due to the shorter operation and hospitalization time and fewer complications of hemorrhage and edema. In contrast, adhesions and re-obstructions are the most frequent etiologies for operative failure [12]. Several studies encouraged to use intraoperative silicone stenting between the nasal cavity and canaliculus to prevent adhesions and stenosis of the nasal ostium [5].

Silicone is an inorganic surgical material and may trigger intranasal and nasolacrimal granulation tissue leading to the development of adhesions and stenosis. Postoperative infections, displacement, and canalicular damage are other possible complications [13]. Prolonged silicone tube intubation has been reported to induce inflammation, infection, and fibrosis [14]. Okuyucu et al. reported three cases of granulation tissues, one case of intranasal synechia and one case of conjunctivitis among 30 patients (all stents were removed at the third month) [15]. Imamoglu et al. detected an inflammatory mass at the lower canaliculus at the second month after surgery. The mass regressed after medical therapy but only after the removal of the silicone tube [16].

Canalicular laceration is one of the main complications of silicone stent intubation and leads to the development of epiphora. Smit and Mourits reported a case series including 13 patients with an untreated monocanalicular laceration. No patients complained of epiphora under basal tear conditions and only three were found to have epiphora under reflex tear conditions [17]. In our study, only one case complained of epiphora due to intranasal synechia and narrow ostium after the removal of the tube. Therefore, we decided to monitor the cases rather than performing canalicular repair.

The incidence of cheese wiring has been reported to be 2.6% for Ex-DCR and 1.5% for En-DCR [18]. Cheese wiring results from the tube being placed under tension and long duration intubation time. Anuar and Gendeh mentioned that wiping the eye when the eyelids are closed may be a cause of cheese wiring. Since the stent loop is immobilized, external wiping of the eyelid pulls the punctum against the fixed stents [19]. Charalampidou et al. recommended early monitoring of the tube within a week of intubation and anticipation of cheese wiring if the tube was too tight [20].

There is still a debate on the extubation time of silicone tubes in the literature. Nuhoglu et al. and Kong et al. suggested removing the tubes before the second month, while Rebeiz et al. stated that tubes should be left in for up to six months [21], [22], [23]. In this paper, we report three cases of nasolacrimal duct obstruction that have been treated by endoscopic dacryocystorhinostomy with silicone tube insertion. The mean indwelling time of silicone tube was 9.3 months, and all the patients had an upper and lower canalicular laceration near the common canaliculus. Despite the case report of Mimura et al. that suggested both the lacrimal system and the silicone tube were tolerant to prolonged intubation, we detected canalicular laceration as a complication of silicone tube stenting in our cases [24].

## Conclusions

In our clinical approach, the removal time for silicone stent is two to four months after operation. Prolonged intubation causes increased nasal inflammation and fibrosis as well as canalicular laceration. Based on the findings of the current study, we recommend removing the silicone tube no longer than four months after the operation.

## Notes

### Competing interests

The authors declare that they have no competing interests.

### Ethical statement

All procedures involving human participants were performed in accordance with the ethical standards of the local ethical committee of Suleyman Demirel University and the 1964 Helsinki declaration and its later amendments or comparable ethical standards. Informed consent was obtained from all individual participants included in the study.
